# The Division of Extramural Research and Training Welcomes Two New Program Administrators

**Published:** 2007-03

Dr. Heather F. Henry joined the Center for Risk Analysis (CRIS) on 10 July as a program administrator for the Superfund Basic Research Program (SBRP). She received a B.S. in Biology from the University of Rochester, which included a year of study in plant-derived medicine at universities in Australia and Ecuador. In 2004, she completed a Ph.D. from the University of Cincinnati, where she was an SBRP trainee participating in numerous multidisciplinary research projects. Henry has recently returned from a Fulbright Postdoctoral Fellowship in Australia studying the role of arbuscular mycorrhizal fungi on arsenate acquisition by native grasses growing on gold mine tailings.

Henry’s primary role has been to initiate the SBRP’s new Individual Research Project Program, which is designed to address specific issues that complement the multiproject research programs, meet high-priority research needs of the SBRP, or tackle issues of emerging concern. The first request for information (RFA), released in November, encouraged the development of innovative approaches to remediate contaminated sediments. Henry’s most recent activity has been to organize the upcoming web-based seminar series “Nanotechnology—Applications and Implications for Superfund,” which will highlight the potential of nanotechnology to support characterization and remediation of hazardous waste sites as well as explore the potential risks of this new class of compounds. For more information on the series, please see **http://www-apps.niehs.nih.gov/sbrp/products/products4.cfm.**

Dr. Daniel Shaughnessy joined the Susceptibility and Population Health Branch (SPHB) on 16 May. He received a BM degree from the Eastman School of Music in Rochester, New York. He completed an MPH and a Ph.D. from the University of North Carolina, Chapel Hill, in 2000 and 2002 respectively.

His research interests are in the molecular mechanisms of antimutagenic compounds, in the role of diet in modulating cancer risk, and in developing short-term markers of effect related to dietary exposures. He has also studied the genotoxic effects of disinfection by-products in drinking water. As a graduate student, Shaughnessy worked with David DeMarini at the U.S. EPA’s Environmental Carcinogenesis Division. He joined Jack Taylor’s lab at NIEHS as a postdoctoral fellow in 2002, where he conducted a human controlled feeding study on the effects of fried meat on DNA damage and the possible inhibition of that damage by other dietary compounds. Results from this work demonstrate that protective dietary components—cruciferous vegetables, yogurt, and chlorophyllin—significantly inhibited DNA damage induced by the fried meat.

Shaughnessy is managing a portfolio of grants related to DNA damage, DNA repair, and mutagenesis. He is also involved in the Exposure Biology Program within the Genes and Environment Initiative, participating in the formulation and implementation of two RFAs aimed at stimulating research to improve measures of early biological responses to common environmental stressors. For more information on the Genes and Environment Initiative, please see **http://www.gei.nih.gov/exposurebiology/index.asp.**

Contacts

**Heather Henry, Ph.D.** | henryh@niehs.nih.gov

**Daniel Shaughnessy, Ph.D.** | shaughn1@niehs.nih.gov

## Figures and Tables

**Figure:**
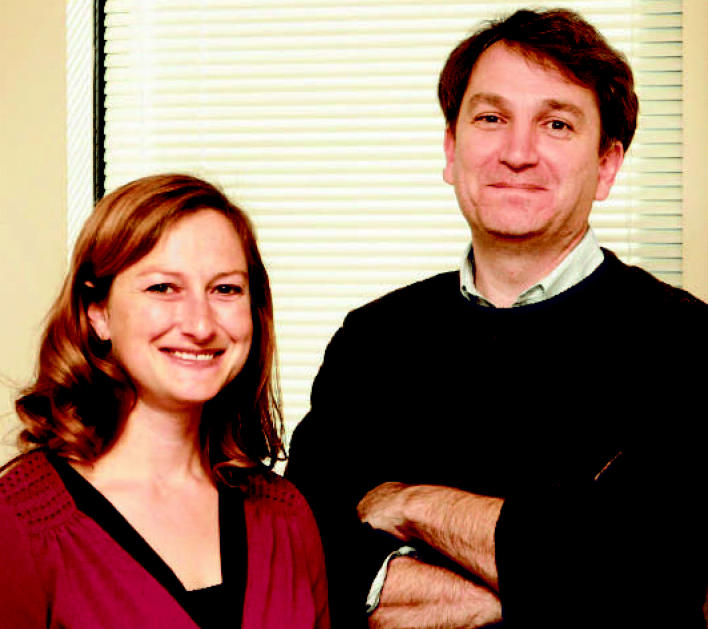
Heather F. Henry and Daniel Shaughnessy

